# The Use of Wood Pellets in the Production of High Quality Biocarbon Materials

**DOI:** 10.3390/ma15134404

**Published:** 2022-06-22

**Authors:** Bogdan Saletnik, Aneta Saletnik, Grzegorz Zaguła, Marcin Bajcar, Czesław Puchalski

**Affiliations:** Department of Bioenergetics, Food Analysis and Microbiology, Institute of Food Technology and Nutrition, College of Natural Science, Rzeszow University, Ćwiklińskiej 2D, 35-601 Rzeszow, Poland; asaletnik@ur.edu.pl (A.S.); gzagula@ur.edu.pl (G.Z.); mbajcar@ur.edu.pl (M.B.); cpuchalski@ur.edu.pl (C.P.)

**Keywords:** wood pellet, pyrolysis, calorific value, explosibility, dust

## Abstract

Biomass is one of the most important sources of renewable energy. One of the most widely used biomass biofuels is wood pellets. It is an economical, homogeneous and easy-to-use raw material. Biomass is used to generate low-emission energy utilizing the pyrolysis process. Pyrolysis allows for higher energy efficiency with the use of commonly available substrates. This thesis presents the results of research on the possibility of using the pyrolysis process to produce high-energy biocarbons from wood pellets. Data on basic energy parameters and explosivity of biocarbon dust were compiled as criteria for the attractiveness of the solution in terms of energy utility. The research used pellets made of oak, coniferous, and mixed sawdust, which were subjected to a pyrolysis process with varying temperature and time parameters. Carbon, ash, nitrogen, hydrogen, volatile substances, heavy metals, durability and calorific value of the tested materials were carried out. The highest increase in calorific value was determined to be 63% for biocarbons obtained at 500 ℃ and a time of 15 min, compared with the control sample. The highest calorific value among all analyzed materials was obtained from coniferous pellet biocarbon at 31.49 MJ kg^−1^. Parameters such as maximum explosion pressure, Pmax, maximum pressure increase over time, (dp/dt)max, and explosion rates, Kst max, were also analyzed. It was noted that biomass pyrolysis, which was previously pelletized, improved the energy parameters of the fuel and did not increase the risk class of dust explosion. The lowest and highest recorded values of Kst max for the analyzed materials were 76.53 and 94.75 bar s^−1^, respectively. The study concluded that the process used for processing solid biofuels did not affect the increase in the danger of dust explosion. The results presented in this article form the basis for further research to obtain detailed knowledge of the safety principles of production, storage, transport and use of these new fuels.

## 1. Introduction

The search for and improvement of alternative energy sources is crucial for both energy and environmental protection, and broadly defined sustainable development [1]. The argument for exploring new technologies, including those using biomass, is the slow depletion of fossil fuel resources: hard coal, lignite, oil and natural gas. Less and less favorable forecasts cause prices of these raw materials to rise on the world market, which, without using new technologies, could lead to conflicts and crises. Another very important aspect that promotes the use of biomass is the widely popularized trend of renewable energy sources. Biomass, by definition, is treated as a low-emission carrier with respect to carbon dioxide (CO_2_). During photosynthesis, plants incorporate into their structures the same amount of carbon dioxide that they release during the combustion process, placing CO_2_ balance in the atmosphere at a zero level [2].

A wood pellet is a type of solid fuel produced for energy purposes. Aimed as a homogeneous and easy-to-use solid biofuel, a compaction process is used in its manufacture, i.e., pelletizing by the pressing of ground wood. Pelletization is an extrusion process based on subjecting fine dry biomass to high pressure and increased temperature, squeezing it through small holes, and pressing small cylinders to the desired length [3,4,5,6,7,8,9]. Wood pellet is characterized by a granule form with a diameter of 6–8 mm and a length of up to 40 mm. Compared with wood chip biomass, pellets have a much higher energy density per unit mass and volume. Pellets are more favorable in terms of storage, handling and transport, and are more homogeneous in terms of physical and chemical properties. Whereas wood chips may be preferred in small facilities, wood pellets are easier to use in larger installations, and in trade and transport over long distances [10,11,12]. Pellets can show significant discrepancy in terms of their quality [13]. The process of drying biomass, its length, moisture level of material, homogeneity, and the origin and type of raw materials for pellet production, directly determine the calorific value of the final product [14,15,16]. Properties of biomass (chemical composition, type and content of extracts, lignin content, water content, particle size, age of the raw material, etc.), conditions of the pelletization process, and possible additives, affect the color of the obtained pellets [17,18,19,20]. There is also a correlation between the color of pellets and their quality; brighter samples obtain better results in terms of mechanical durability, bulk density and heating values [18].

Sweden, Germany, Austria and Latvia are the main countries producing granules/pellets in the European Union. The possibility of using this material in Europe is regulated at the state level. Implementation of regulations such as tax exemptions, subsidies and biomass sustainability policies have contributed to the increase in production and use of wood pellets. In many European countries such as Belgium and the Netherlands, wood granulates are used to a very large extent in power plants. Pellet is widely used in thermal energy production in many individual households as well as in the industrial sector. The past decade marks a traceable increase in the development of the pellet market in Europe with regard to small heating systems [21,22]. The use and production of pellets in Poland against the background of the European Union is only slightly lower. The variety of materials and relatively easy technological process affects the continued interest in the pellet market in Poland.

The primary factor that characterizes fuel as useful in energy production is its energy value. The average calorific value of pellets is in the range of 15 to 18 MJ kg^−1^ [23]. As interest in wood pellets has increased, methods have been developed to modify its basic energy parameters. For this purpose, biochemical and thermochemical methods have been used, e.g., torrefaction and pyrolysis. The literature has also reported on the possibility of introducing various types of additives into pellets, such as glycerin, cooking oil and starch. These modifications have been conducted at the production stage or in the form of post-production treatment [23,24]. 

One possibility of using biomass to acquire low-carbon energy is to use it in the pyrolysis process. The use of this technology allows for higher energy efficiency using commonly available substrates. The main aspect in favor of using the pyrolysis process is its versatility. In addition to processing raw materials into energy carriers, it also produces raw material recycling, enabling the recovery of materials used in the production of a particular product. The use of biomass pyrolysis process to produce biocarbons contributes to local technological development and increases public environmental awareness, through the use of renewable energy sources. A variety of substrates such as plant and waste biomass can be used in the production of biocarbon. This allows for the management of various types of waste or plant parts that are unattractive in terms of food or economy. The main advantages of the pyrolysis process are to increase the calorific value of the materials used and the reduction in their bulk density [3,4]. The bulk density and calorific value together determine the energy density of the pellets [25]. Pellets have a much higher energy density per unit of mass and volume compared with raw biomass and wood chip biomass [13].

The processing of plant biomass associated with obtaining fractions of small sizes is associated with the danger of heavy dustiness of indoor air. Hazards associated with dust explosion or self-ignition may arise from processes used in bioenergy technologies. Numerous studies and procedures developed so far have undoubtedly increased safety in this sector, but it seems appropriate to carry out further studies to eliminate the risk of dust explosion. The dynamics of the development of the sector of large bioenergy installations, small prosumer installations and the variety of available solid biofuels directs the need to improve existing processing schemes. The use of equipment for milling, crushing or material handling processes can affect the occurrence of dust explosion hazards. Considering the use of such processes in industrial plants, the likelihood of such dangers remains a constant threat [26,27,28,29,30].

Reaching a minimum explosion concentration indoors with spreading fine dust can lead to an immediate explosion. The explosion happens when five basic elements are present at the same time: combustible dust, oxygen in air, ignition, dust particulates dispersion, and confinement of dust clouds. Very serious, even fatal, consequences may happen, when those five conditions meet. Accidents have been reported in relation to wood pellet auto-ignition and consequent fire [31]. The dusts that carry the risk of explosions are formed during the processing of wood materials, paper products, textile and food production, metalworking, and fossil fuel extraction. However, it should be emphasized that dust explosion is conditioned by certain parameters, i.e., the “explosivity pentagon”. The following conditions must be met: space limitation, mixing of fuel and oxidizer, ignition source, oxidizer, and fuel in the form of dust. Improper operation and use of material processing equipment, high temperature of plant components, and gases generated, e.g., during biomass treatment, may also constitute an elevated risk factor for an explosion hazard. The transformation of lignocellulosic materials, e.g., through grinding and crushing, may also affect the formation of electrostatic discharges and thus increase the risk of explosion [32,33,34,35,36,37]. The risk of dust explosion is equally high during production, transport and storage of biomass fuels, due to the formation of a high concentration dust cloud. Dust explosions, unfortunately, almost always lead to serious accidents and financial loss. What is important in this regard is appropriate safety rules regarding contact with powdered biomass. In order to assess the possibility and minimize the risk of dust explosion, the basic parameters of biomass explosiveness must be known [38,39,40]. An understanding of the influence of the interactions among lignocellulosic biomass components can aid in preventing and controlling dust explosions. A higher fraction of cellulose enhances the mixture’s weight loss peak intensity and explosion pressure. An increase in hemicellulose improves the explosion rate of pressure rise. An increase of lignin leads to an increase in solid residue and a decrease in explosion pressure. The explosion pressure of the mixture is mainly determined by the promotion and inhibition effect of cellulose and lignin, respectively, and the rate of pressure rise is mainly affected by hemicellulose content [41].

The crucial importance of plant biomass, its conversion methods and biocarbon materials in both ecological, economic and energy aspects, is emphasized by the importance of continuous research and the need to increase knowledge in this sector.

Over the last decade, research has been carried out on the thermal behavior, properties of biochar, reaction mechanisms, and the kinetics of pyrolysis of biomass pellets. Yan et al. [42] investigated the fuel properties of biochar pellets from Chinese fir sawdust pellets at various temperatures (400, 450, 500, 550 and 600 °C), heating rates (2, 6 and 10 min^−1^) and residence times (60, 120 and 180 min). Scientists reported that biochar pellets had the highest calorific values, and higher compression and breaking resistance, at 550 °C. The best properties of pellets were recorded for the heating rate of 28 min^−1^ and the residence time of 120 min. Zhou et al. [43] analyzed the pyrolysis of granular municipal waste and found that the carbonization efficiency decreased significantly with increasing temperature from 450 to 900 °C, due to the high content of plastic groups at high decomposition temperatures. Basu et al. [44] investigated the effect of torrefaction on the density and volume of coarse-grained biomass particles. Scientists noted that radial contraction was 3–4%, a reduction in the longitudinal direction was 6.5–8.8%, and that mass efficiency decreased as torrefaction increased. Chen and Lin [45] analyzed pyrolysates from oil palm fiber granules in an atmosphere of nitrogen and carbon dioxide. They noted that pellets could be used as a raw material and CO_2_ as a pyrolysis carrier gas, which had the advantage of reducing the reactor volume and allowing the use of CO_2_. Ghiasi et al. [46] discussed the pros and cons of torrefaction after densification, and densification after torrefaction. The results showed that the compaction and torrefaction of wood chips was energy-saving. Xing et al. analyzed the pyrolysis of corn straw pellets at different temperatures (400, 450, 500, 550 and 600 °C) at a 10 °C min^−1^ heating rate and a residence time of 30 min. The scientists studied the characteristics of biochar pellets, in particular their elemental composition, hydrophobicity and mechanical resistance. The results showed that the mass and energy efficiency of the biochar pellets decreased at elevated temperatures. Meanwhile, the higher calorific value of biochar pellets increased with increasing temperature. In addition, the scientists reported that biochar pellets showed good hydrophobicity, which had a positive effect on their storage and transport, although their mechanical resistance decreased [47].

Learning about the nature of changes in the chemical structure of solid biofuels, and improving the methods of their processing, storage and transport, undoubtedly allows for the development of appropriate standards of conduct and environmental protection. It seems reasonable to develop detailed characteristics with a particular focus on the processes and related safety rules. These types of studies can provide a theoretical basis and technical support for the prevention and control of biomass dust explosions in industrial processes. 

The aim of this research was to evaluate the possibility of using wood pellets for the production of fuels with increased energy value, using the pyrolysis process. An additional objective was to determine the basic parameters of explosiveness of the tested materials.

## 2. Materials and Methods

### 2.1. Research Object

In this study of the assessment of the conditions for the pyrolysis of wood pellets, biofuels available commercially on the Polish market were used. In the case of vegetable biomass, fruit biomass was used for selected properties of biochar. The test material was comprised of different types of pellets (diameter of 6 mm) from a manufacturer from Podkarpackie Voivodeship (Poland), produced in 2021:

- oak sawdust pellet;
- coniferous sawdust pellet;
- mixed pellet, i.e., coniferous and deciduous (70% from coniferous sawdust, 30% from deciduous sawdust).

The exact forest species, or mixture of species, used as the feedstock for the produced pellets was not provided on the packaging. The packaging also did not contain information on the exact time period of the production of the pellets, nor information on the transport and storage conditions of the product. The water content declared by the manufacturer for the purchased pellets was 5–6%. The test material was delivered to the laboratory in the form of commercially available 15 kg packs.

### 2.2. Pyrolysis Process

**Figure 1:**
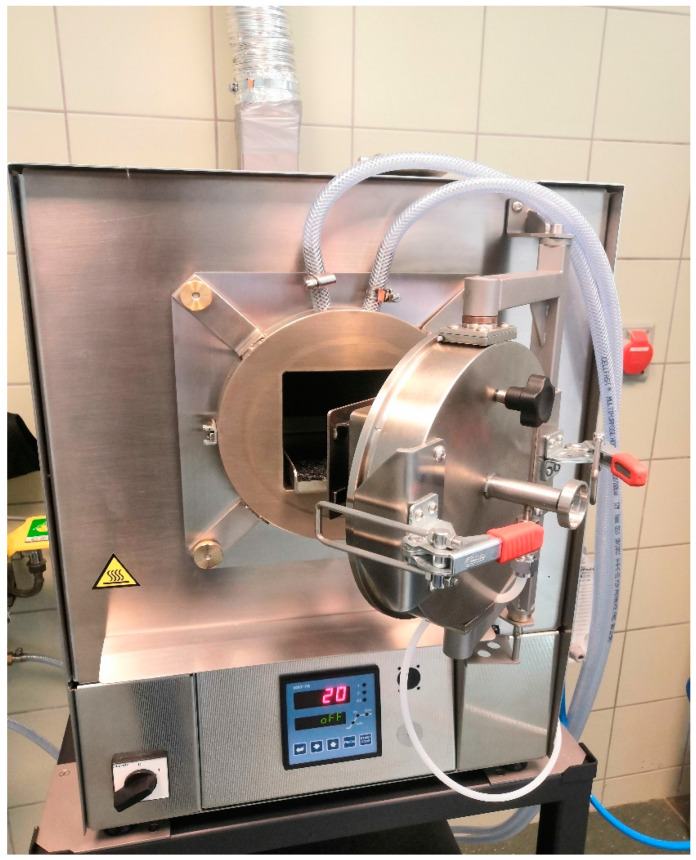
Pyrolysis retort furnace.

The pyrolysis process was carried out using a retort furnace FCF 2R (CZYLOK, Jastrzębie-Zdrój, Poland) designed for heat treatment in an atmosphere of inert gas, equipped with a post-process gas cooler with water well (Leco, St. Joseph, MI, U.S.A.) (Figure 1).

**Figure 2:**
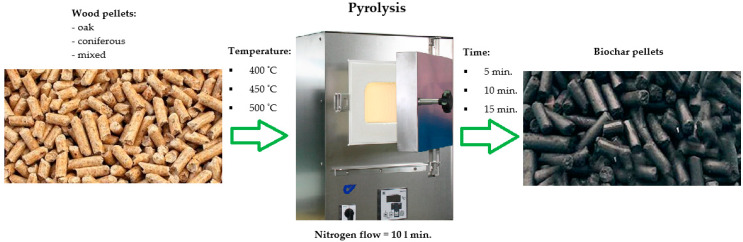
Diagram of the process used.

Pyrolysis tests of the test pellet families were carried out at temperatures of 400, 450 and 500 °C and a maintenance time of 5, 10 and 15 min in a nitrogen atmosphere of 99.99% purity with a gas flow of 10 L/min (Figure 2). Then the obtained pyrolysates were sifted through a sieve with a hole diameter equal to 1 mm. In order to remove potential contaminants (mechanical impurities, i.e., chips, sawdust, dust, etc.), the samples were rinsed several times with distilled water and then dried for 12 h (temperature 80 °C).

### 2.3. Analysis of Samples

Basic physicochemical parameters of the analyzed materials were determined, e.g., the total content of carbon, ash, nitrogen, hydrogen, volatile substances, and calorific value, using a thermogravimeter LECO TGA 701, an elementary composition analyzer TrueSpec LECO CHN (Leco, St. Joseph, MI, U.S.A.), and a LECO AC 500 isoperibolic calorimeter (Leco, St. Joseph, MI, U.S.A.). 

**Figure 3:**
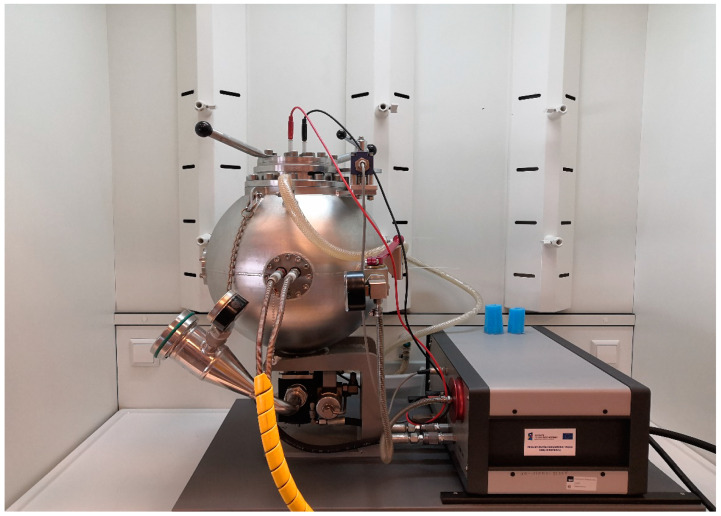
Explosivity Analyzer KSEP 310.

Dust explosiveness analyses were carried out using the KSEP20 device equipped with a control unit KSEP 310 (Kuhner AG, Basel, Switzerland). The device featured a test chamber in the form of a ball with a volume of 20 dm^3^. Explosion heat dissipation and the provision of thermostatically controlled test temperatures was provided by a water jacket (Figure 3).

The analyzed dust was dispersed under pressure using an inlet valve, which was opened and closed pneumatically. The ignition source was designed as two chemical igniters with an energy of 5 kJ each, located in the central part of the sphere. The course of the process parameters was recorded using Kistler pressure piezoelectric sensors. As a result of the analyses, the maximum explosion pressure, Pmax, was determined as the highest recorded explosion pressure of the combustible mixture in the form of combustible material with air. This parameter, along with the value of the maximum pressure gain over time, (dp/dt)max, was used to determine the explosiveness class, Kst max. This parameter is a determinant of European standards, which defines the division of combustible dust according to EN14034 [29]. The parameter was estimated from the equation:

(1) $$K\max=K\mathrm{st}=\sqrt[{3}]{V(\frac{dp}{dt}})\max=0.271(\frac{dp}{dt})\max[{\mathrm{mbars}}^{-1}]$$

where:

Kst max—explosivity index;

V—volume of test chamber;

(dp/dt)max—indicator of maximum explosion pressure gain.

The value of the explosivity index was classified according to the values shown in Table 1, where class St1 means a material that exhibits low susceptibility to explosiveness, class St2 means a material exhibiting medium susceptibility to explosive hazard, while class St3 means a material highly susceptible to explosive hazard.

Samples of biomass and biochars were subjected to laboratory analyses using current analytical standards (Table 2).

Analyses of the contents of ash and volatile substances in the samples were performed using a thermogravimetric method, with a TGA 701 apparatus from LECO (LECO Corporation, Saint Joseph, MI, U.S.A.). The contents of total carbon, hydrogen and nitrogen were tested using a TrueSpec CHN analyzer from LECO (LECO Corporation, Saint Joseph, MI, U.S.A.). An AC500 calorimeter from LECO (LECO Corporation, Saint Joseph, MI, U.S.A.) was used to determine the calorific value of the materials analyzed.

Mechanical durability was tested using a Tumbler 1000 apparatus. The part subjected to the test, (mE) 500 ± 0.1 g, was placed in the drum and them tumbled for 10 min with a speed from 500 rotations/min to 0 rotations/min. Subsequently, the sample was manually sifted through a sieve with 3.15 mm openings. Subsequently, the sample remaining on the sieve was weighed (mA). The test was performed in triplicate for each variant. The mechanical durability of pellets (DU) was calculated following the formula:

DU = mA/mE × 100

where:

DU—mechanical durability of pellets;

mA—pellet weight following the test (g);

mE—pellet weight before the test (g).

The measurement of heavy metals content was performed on an ICP-OES spectrometer, Thermo iCAP Dual 6500 with horizontal plasma, and with the capacity of detection being determined both along and across the plasma flame (radial and axial). Before measuring each batch of 10 samples, the equipment was calibrated with the use of certified Merck models. The measurement result for each element was adjusted to account for the measurement of elements in the blank sample. In each case, a 3-point calibration curve was used for each element, with optical correction in applying the method of internal models, in the form of yttrium and ytterbium ions, at concentrations of 2 mg/L and 5 mg/L, respectively. The analytical methods were validated using two independent tests. The detection threshold obtained for each element was not lower than 0.01 mg kg^−1^.

### 2.4. Names of Tests

For further identification, biomass samples were described using symbols depending on the type of material, temperature, and duration of the pyrolysis process:

OP—oak sawdust pellet;

CP—coniferous sawdust pellet;

MP—mixed pellet (coniferous and deciduous sawdust);

0—thermally unprocessed material;

1—pyrolysis (temp 400 °C; 5 min);

2—pyrolysis (temp 400 °C; 10 min);

3—pyrolysis (temp 400 °C; 15 min);

4—pyrolysis (temp 450 °C; 5 min);

5—pyrolysis (temp 450 °C; 10 min);

6—pyrolysis (temp 450 °C; 15 min);

7—pyrolysis (temp 500 °C; 5 min);

8—pyrolysis (temp 500 °C; 10 min);

9—pyrolysis (temp 500 °C; 15 min).

For instance, OP0—oak sawdust pellet without heat treatment, and OP1—oak sawdust pellet subjected to a pyrolysis process at 400 °C and a maintenance time of 5 min.

### 2.5. Statistical Analysis

The effects of the experimental factors reflected by the relevant parameters, and the relationships between these, were examined using analysis of variance (ANOVA) by means of the Duncan test. Statistica 12 software was applied to compute the statistical analyses. A significance threshold of ≤0.05 was set for all analyses. The data were analyzed separately for each type of pellet [56,57].

## 3. Results

### 3.1. Oak Sawdust Pellets and Biocarbons

Table 3 compiles data on the percentage of total nitrogen, total carbon, hydrogen, ash, and volatile substances in thermally unprocessed oak pellets and its biocarbons produced thereof, and the calorific value of the tested materials. The level of total nitrogen in the analyzed samples was below 0.04%—the result below the detection limit of the device. The level of total carbon varied depending on the degree of processing of the material. The sample of unprocessed pellets had a total carbon content of 51.46%. Values in the range of 75.25–81.41% were obtained for biocarbons formed in the pyrolysis process of oak pellets. The lowest value was noted for the pyrolysis process carried out at 400 °C and a time of 5 min, and the highest for parameters 500 °C and 15 min. The hydrogen content of the tested samples also varied depending on the parameters used for the pyrolysis process. The level of hydrogen content for the resulting biocarbons was in the range of 3.19–4.33% and was significantly statistically lower than in the control sample, i.e., unprocessed pellets.

The general ash content for the resulting biocarbons was several times greater than the control sample (0.53%) and was in the range of 3.75–6.63%. According to the European Union standard, the ash content in premium wood pellets should be less than 0.7%, and PFI defines this parameter as less than 1% [23]. The ash content in the obtained char was higher than the prescribed standards. The highest ash content was characterized by a sample formed at a temperature of 500 °C and a time of 15 min. The lowest total ash content was recorded for material formed at the lowest temperature and shortest time. Each of the resulting biocarbons significantly statistically differed in their ash content from the sample of unprocessed pellets. There were also statistically significant differences between samples formed at 400 and 500 °C. The volatile substance content for the resulting biocarbons, in turn, was significantly lower than in the control material (81.73%) and was in the range of 28.34–37.05%. The lowest content of volatile substances was characterized by biocarbon produced in the highest applied parameters of the pyrolysis process.

The durability of tested biocarbons ranged from 44.92 to 56.14%, which was below the untreated sample value of 99.12%. It was observed that durability of the biocarbon pellets increased with increasing pyrolysis temperature but remained lower than the durability of the raw pellets.

All the tested samples did not contain arsenic and cadmium—the result below the detection limit (0.01 mg kg^−1^). Contamination of pellets and biocarbons with lead ranged as follows: 0.11 mg kg^−1^ for the control sample, and 0.16–0.34 mg kg^−1^ for materials after pyrolysis. The highest increase of Pb content was noted at the highest temperature of pyrolysis. At the same time, it was found that the change in the temperature of the process was significantly affected by this heavy metal content.

The calorific value of the thermally unprocessed oak sawdust pellet was 18.27 MJ kg^−1^. The use of the pyrolysis process allowed a significantly higher calorific value to be obtained, which ranged from 27.22 to 30.45 MJ kg^−1^. The highest calorific value was obtained after applying a temperature of 500 °C and a maintenance time of 15 min. Each of the resulting biocarbons differed significantly statistically from the sample of unprocessed pellets with respect to this parameter. There were no statistically significant differences in calorific value between biocarbons obtained under different pyrolysis conditions.

### 3.2. Coniferous Sawdust Pellets and Biocarbons

Table 4 contains data showing basic energy parameters of coniferous pellets and the biocarbons produced therefrom. As in the case of oak pellets, the nitrogen content was not recorded for the tested untreated or biocarbon materials. By contrast, statistically significant differences in the carbon and hydrogen content of the coniferous pellet biocarbons, compared with the unprocessed biomass, were noted. The control test, i.e., coniferous pellets, showed a total carbon content of 53.25%. The content of this element in the obtained pyrolysates increased significantly statistically and was in the range of 75.98–85.21%. The lowest value was recorded for the pyrolysis process conducted at 400 °C and a time of 5 min, and the highest for the process carried out at 500 °C and a time of 15 min. The level of hydrogen content in the resulting biocarbons varied depending on the thermal degree of processing of the material. The baseline hydrogen content was 6.21%, while the use of the pyrolysis process reduced this parameter to 3.02–4.49%. The highest value of the test parameter was characterized by biocarbon obtained at 400 °C and a time of 5 min.

The general ash content of the thermally unprocessed coniferous pellet sample was 0.37%. The ash content of produced biocarbons was several times higher, in the range of 3.16–4.73%. Again, the highest content of the test parameter was characterized by the sample formed in the highest pyrolysis parameters used. Each of the resulting biocarbons differed significantly statistically from the sample of unprocessed pellets with respect to this parameter. The percentage of volatile substances in unprocessed coniferous pellets was 82.81%, while in the obtained biocarbons, there was a decrease to 29.24–39.62%. There was a statistically significant relationship between the increase in the temperature of the pyrolysis process and the decrease in volatile substances content in the produced biocarbons.

The durability of coniferous sawdust pellets was 98.54%. The use of the pyrolysis process decreased this parameter, in the range of 44.34–55.98%. The pellets produced at the highest applied temperature were characterized by higher resistance, compared with other biocarbons.

In coniferous sawdust pellets and the biocarbons produced therefrom, arsenic and cadmium contents were not detected. Contamination of coniferous pellets and biocarbons with lead was greater than for oak sawdust, which ranged as follows: 0.23 mg kg^−1^ for the control sample, and 0.32–0.48 mg kg^−1^ for materials after pyrolysis.

The results of the analyses showed that the calorific value of pellets for the production of which coniferous sawdust was used was 19.31 MJ kg^−1^. The calorific value of coniferous pellet biocarbons increased statistically to 28.88–31.49 MJ kg^1^. Again, the highest increase in the test parameter was attributed to the highest temperature and the longest time for conducting pyrolysis; at the same time it was not found that the change in the temperature of the process was significantly affected by the change in the test parameter.

### 3.3. Mixed Sawdust Pellets and Biocarbons

As in the variants discussed above, no nitrogen content was recorded in the analyzed mixed pellets (coniferous and deciduous) or the biochars derived from them (Table 5). Subjecting mixed pellets to pyrolysis allowed the total carbon content to increase from 52.52% to values in the range of 75.67–83.32%. In the case of hydrogen content, it was reported that in brews with an increase in temperature and time of pyrolysis process, the content of this element decreased from 6.16% for the control, to 3.24% in the biocarbon.

The ash content of mixed pellets (0.31%) was similar to that of coniferous pellets. Prepared biocarbons were again characterized by several times higher ash content in the sediment for the control test. The maximum recorded content was 4.95%. The content of volatile substances in the control, i.e., mixed pellets (82.66%) and biocarbons produced (28.97–38.51%), was very similar to that of the previously discussed types of tested materials.

The durability of the control sample was 98.87% and mixed biocarbon pellets was in the range of 44.51–56.07%, which was very similar to the oak and coniferous sawdust pellets and biocarbons produced therefrom.

Arsenic and cadmium contents were not detected. The lead content for the resulting biocarbons was definitely greater than the control sample (0.21 mg kg^−1^) and was in the range of 0.29–0.44 mg kg^−1^. The highest value of Pb content was characterized by biocarbon obtained at 500 °C.

Coniferous leaf pellets had little lower calorific value than coniferous pellets and were slightly higher than oak pellets, with a value of 19.13 MJ kg^−1^. The pyrolysis process increased the calorific content of the material to a maximum value of 30.73 MJ kg^−1^. Once again, there were no statistically significant differences between held biocarbons for this test parameter.

### 3.4. Dust Explosion Parameters

The following table summarizes the results of tests on the level of lower dust explosivity limit, maximum pressure rise rate ((dp/dt)max), and maximum explosion pressure (Pmax), of dust from oak, coniferous, mixed pellets and their pyrolysates (Table 6). The results of the analyses represent a similar relationship of variations in parameter values for the tested materials. The mean Pmax value of the analyzed pellet dust was 7.94 bar. In the results of the pyrolysis process, this value increased to a maximum level of 11.6 bar for dust from coniferous sawdust pellets subjected to pyrolysis. A strong correlation of the increase in maximum explosion pressure was observed with respect to increasing the temperature–time parameters of pyrolysis. Very similar dynamics of change were also noted for the maximum pressure build-up rate parameter. Dust from non-thermally processed pellets reached the lowest values of this parameter relative to pellets after the pyrolysis process, for which the values increased as the parameters of the process were increased. The highest value (dp/dt)max of 349.64 bar s^−1^ was characterized by pyrolyzed coniferous pellet dust at 500 °C and 15 min. There was also a variability in the level of the lower explosive limit of dust from non-thermally processed pellets relative to pyrolysis pellets. The LEL level of dust from oak and mixed pellets decreased when the pyrolysis process used was 450 °C/5 min or higher, and for coniferous pellets at 450 °C/10 min or higher.

**Figure 4:**
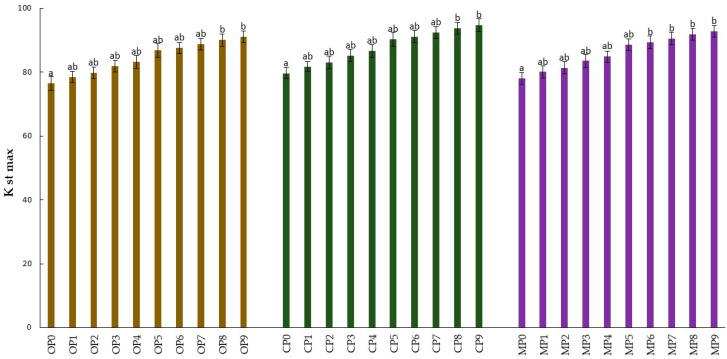
Dust explosivity index of oak, coniferous, mixed pellets and produced biocarbons. Statistically significant differences between values are marked by different letters (*p* ≤ 0.05).

The test directly describing the risk of dust explosion is the explosivity index, Kst max, calculated on the basis of the standard [39]. The analysis of the value of this parameter classified the thermally unprocessed oak, coniferous pellets and their mixture, as well as the thermally processed forms obtained from them, into the first class of dust explosion hazard (St1)—a material not susceptible to explosiveness. The explosivity rate for pellets of oak, coniferous and mixed sawdust was 76.53, 79.64, and 78.05 bar s^−1^, respectively. There was an increase in this parameter for the obtained biocarbons with an increase of the temperature range and the duration of the pyrolysis process. In each case analyzed, the highest values were obtained for pellets pyrolyzed at 500 °C and a time of 15 min. The highest explosivity index value among the analyzed materials was characterized by coniferous pellets reaching 94.75 bar s^−1^ (Figure 4).

**Figure 5:**
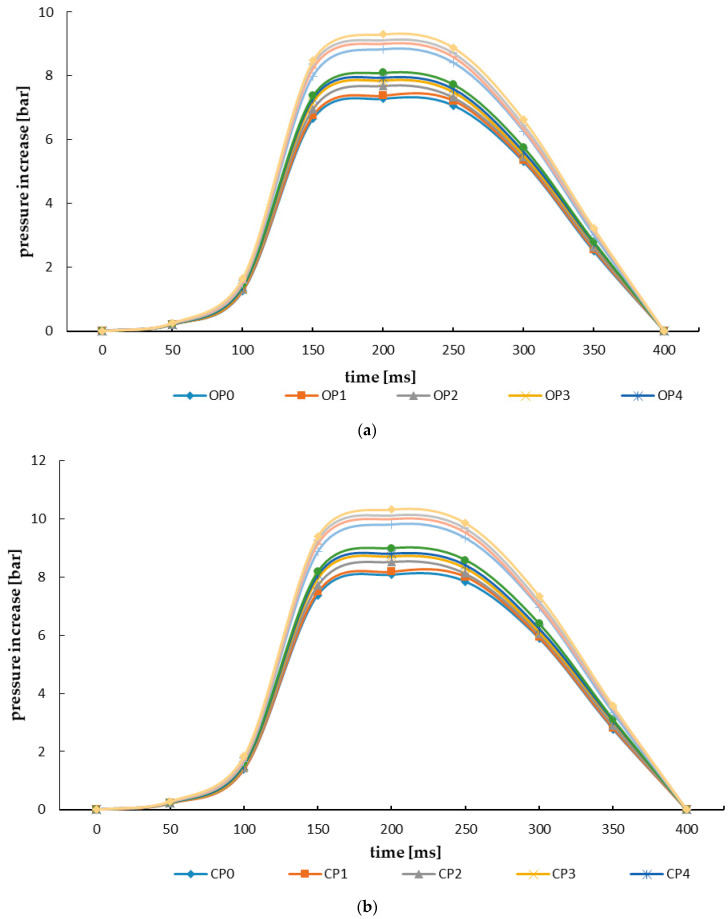

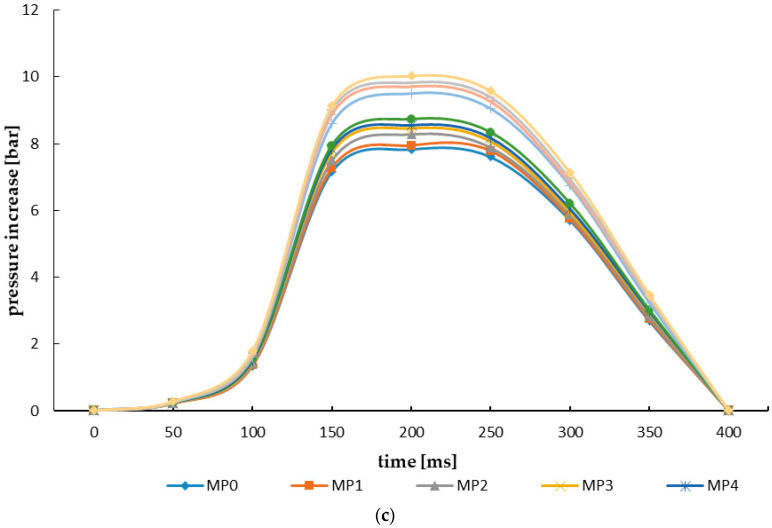
Distribution of dust explosion pressure of pellets and biocarbons produced therefrom: (**a**) oak sawdust material, (**b**) coniferous sawdust material, (**c**) coniferous–deciduous sawdust material.

Trends of changes in the flow of explosion pressure with respect to the test material per unit time are presented in Figure 5. As a result of the analysis, similar trends of changes in explosion pressure were observed, which were significantly influenced by the differentiation of the applied thermal treatment conditions, with a significant indication of an increase in process temperature. The maximum explosion pressure of oak, coniferous and mixed sawdust pellets, and the biocarbons produced from them, was recorded at 200 ms upon the initiation of the explosion. There was also a relationship determining the increase in dust explosion pressure with increasing parameters of the pyrolysis process (Figure 5).

## 4. Discussion

Presently, there are many processes aimed at improving the properties of various types of materials for energy suitability. Biomass resources have high moisture content and low bulk energy densities, and exhibit propensity to decay during storage, and thus, their use is limited. Several methods, such as pelletization and pyrolysis, are used for overcoming these disadvantages and other related problems [47]. Pellets may vary in quality. The calorific value of pellets depends on the drying time of the biomass during the treatment process (humidity level), the consistency of the granulate, and, most of all, on the source and type of raw material. A satisfactory final moisture content of wood pellets should be within 5–10%. The higher the water content, the lower the calorific value of the pellets. The ash content should not exceed 1.5%. The binder in wood pellets is lignin, therefore, the more there is, the better the quality of pellets that will be produced [23].

Application of pyrolysis consists of obtaining more favorable energy parameters, and, above all, raising the calorific value, which is one of the most important factors determining the suitability of fuel. It is important to remember to control the quality of the fuels obtained, in particular, in terms of their safe use, storage and transport.

Three different types of pellets from one of the producers from Podkarpackie Voivodeship were tested in the study: oak pellets, coniferous pellets and mixed pellets. Each was subjected to a pyrolysis process at three different temperatures of 400, 450 and 500 °C, and times of 5, 10 and 15 min.

No total nitrogen content was recorded in the tested materials. The pyrolysis process did not affect the change in this parameter. Similar relationships were obtained by Yuan et al. (2013), who subjected sewage sludge to the pyrolysis process, and showed no increase in overall nitrogen levels in their study [58]. Other results were obtained in studies by Al-Wabel et al. (2013), who by subjecting waste from the plant production of Sęczowina (*Conocarpus*) to pyrolysis, observed an increase in nutrients, including nitrogen [59]. In turn, they showed a significant increase in the total carbon content of the resulting pyrolysates relative to the starting material by an average of 30%. The highest percentage carbon content was characterized by samples subjected to the highest temperature, i.e., 500 °C, and the longest time of 15 min. The highest content of total carbon in biocarbons formed from oak, coniferous, and mixed pellets, was as follows: 81.41, 85.21, and 83.32%, respectively. Of the samples analyzed, the highest carbon values after processing resulted for coniferous pellets. Similar results were obtained in their research by Ronsse et al. (2012), examining the effect of pyrolysis conditions on pine wood, wheat straw, and green waste, on the amount of obtained elements, and the minimum total carbon. The authors subjecting pine wood, wheat straw, green waste and dried algae to the pyrolysis process observed that the highest total carbon content was obtained at the highest temperature, i.e., 750 °C, and the longest sample roasting time, i.e., 60 min. Green waste samples had the highest carbon content at 98.1% [60]. In research by Elnour et al. (2019) regarding the effects of pyrolysis temperature on the microstructural evolution of biocarbon and physicochemical properties of the resulting biocarbons, the authors undertook studies on lignocellulosic biomass. The temperature of pyrolysis was in the range of 300–700 °C. The highest values of total carbon were obtained at 600 °C, i.e., 74.76% [61]. Angin (2013) obtained similar relationships in his research, studying the effect of pyrolysis temperature and heating rate on yield and physicochemical and morphological properties of biocarbon obtained from safflower (*Carthamus tinctorius*). His results confirmed that the efficiency and quality of biocarbon mainly depended on the temperature used. The highest total carbon content (80.70%) was obtained at 600 °C [62]. Comparable results were obtained by Bajcar et al. in their studies (2018), analyzing the relationship between roasting parameters and physicochemical properties of products obtained from different types of biomass used in the research: wheat straw, rapeseed and energy willow (*Salix x Viminallis*). The highest carbon value was recorded at the highest temperature, i.e., 300 °C, for rapeseed straw (58.5%). The results demonstrated the dependence of the carbon content of the obtained biocarbon samples on the temperature of the process conducted [63].

Analyzing the obtained results, it was found that the highest percentage of hydrogen content was recorded for unprocessed pellets, at 5.96, 6.16, and 6.21%, for oak, mixed and coniferous pellets, respectively. The dependence that characterized all the tested materials was a decrease in hydrogen content with an increase in the temperature of conducting the pyrolysis process. It was also possible to note the correlation of hydrogen content until pyrolysis was completed, i.e., a decrease in the content of the analyzed element along with the prolongation of the thermal treatment. Similar results in their research work were obtained by Elnour et al. (2019), studying the influence of the temperature of the pyrolysis process on the microstructural evolution of biochar and its physicochemical properties. The authors used date palm biomass in their study. The hydrogen content of the biocarbons decreased as the temperature increased; for the lowest temperature (300 °C) it was 3.82% and for the highest temperature (700 °C) it was 0.9% [61]. Comparable results were also achieved by Al.-Wabel et al. (2013) who, when studying biomass waste, saw a significant decrease in hydrogen content with increasing temperature. Their studies were conducted in the range of 200 to 800 °C [59].

The studies also showed the dependence of the ash content on the temperature of the process, observing a significant increase in this parameter in the obtained biocarbons. The highest ash content among test controls was characterized by oak pellets at 0.53%. The highest values of the analyzed parameter were obtained for samples of pyrolysates formed at the longest time and highest temperature, at 6.63, 4.95, and 4.73%, for oak, mixed, and coniferous pellets, respectively. The determining factor in the content of ash parts was also the time of thermal treatment, there was a significant increase in the test parameter depending on the length of the process. The literature on the subject provides similar dependencies. Jin et al. (2016) conducted studies on dried raw sludge at temperatures ranging from 400 to 600 °C, to determine the effect of pyrolysis temperature on the properties and environmental safety of heavy metals in biocarbon derived from municipal sewage sludge. They recorded a significant increase in ash content conditioned by an increase in temperature [64]. In turn, Babinszki et al. (2020) studied the process of torrefaction using the example of the aquatic fern (*Azolla filiculoides*). The process was conducted at three different temperatures, i.e., 260, 280 and 300 °C, in 15 min, with an increase in ash content between control sample and pyrolysates [65]. Close results were also obtained by Bajcar et al. [63] in their study.

The primary reported relationship resulting from the study of volatile substance content in pellets and biocarbons was a decrease in this parameter relative to the increase in applied temperature and the duration of the pyrolysis process. The difference between the unprocessed pellet control sample and pyrolysates averaged more than 40%. Research led by Zhao et al. (2019) confirmed that the volatile content decreased with increasing temperature [66]. Pehlivan (2017) noted, in turn, that volatile components could be removed in the form of gases, which affected the decrease in the volume of carbonizate obtained [67]. In their research thesis, Zhang et al. (2015) observed a similar relationship by studying the effect of pyrolysis temperature and heating time on the physicochemical and morphological properties of biocarbon obtained from straw and lignosulfonate. The authors noted a decrease in hydrogen content as well as a decrease in the level of volatile substances in the obtained biocarbons, with increasing temperature [68].

The studies determined a statistically significant increase in the calorific value of materials subjected to the pyrolysis process. Characterizing pellet samples in unprocessed form, the highest calorific value was characterized by the coniferous pellet sample, at 19.31 MJ kg^−1^. For mixed pellets, this value was 19.13 MJ kg^−1^, while for oak pellets it was 18.27 MJ kg^−1^. The use of the thermal modification process of all pellets tested allowed an average increase in calorific value, relative to the control sample, of 56%. The parameters of pyrolysis that allowed the highest calorific value to be obtained for biocarbons from oak, coniferous and mixed pellets, proved to be at temperature 500 °C and time of 15 min. However, no statistically significant changes in the test parameter between the applied thermal treatment options were noted. Additional heat treatment of pellets in the form of pyrolysis will increase the cost of the material, but seems to be a beneficial solution in terms of energy value, transport and storage of biomass. The presented method is a solid foundation for further research. Khalid Rafiq et al. (2016) tested maize straw to assess the effect of pyrolysis temperature on the properties of the obtained products. The research was conducted at three temperatures, i.e., 300, 400, and 500 °C, and the process duration was up to 2 h. The authors recorded an average increase in the calorific value of pyrolysates relative to the unprocessed sample, at 20% [69]. Close results were obtained by Santos et al. (2020) studying the effect of pyrolysis temperature on the properties of products made from sugarcane pomace and oatmeal scales. The tests were carried out at three temperatures (400–500 °C) and the fuel produced at the highest temperature had calorific values of 33.4 and 33.5 MJ kg^−1^ for bagasse of sugar cane and oat husks, respectively [70]. The dependence of the increase in calorific value with increasing temperature of the pyrolysis process was also confirmed by studies by Ahmad et al. (2020) on the quality of coconut shell biocarbons. The increase in process temperature from 250 to 450 °C increased calorific value from 25.99 to 29.54 MJ kg^−1^ [71]. Similar relationships were documented in Sarkar and Wang’s study (2020) on the process of making and evaluating biocarbons from coconut shells. The authors determined the trend of increased calorific value from 28.1 to 30.6 MJ kg^−1^ with increased pyrolysis temperature from 400 to 800 °C [72]. Poskart et al. (2018) noted similar dependencies in their results during research on the process of torrefaction of Pennsylvania scales at temperatures of 250, 275, 300, 325, and 350 °C, and a time of 20 min. The authors reported an increase in calorific value on average for all pyrolysates by 39.4% over the control sample [73]. According to Arouse et al. (2021), pellets made of softwood biochars characterized a calorific value of 31 MJ kg^−1^ [74].

Durability can be used to predict the ability of pellets and biocarbon fuels to remain intact during transport and storage. The durability of biocarbons were lower than raw pellets. The durability of all tested biocarbons ranged from 44.34 to 56.14%, which was significantly below the control sample’s value (average 98.84%). It was found that the durability of biocarbon pellets increased with increasing pyrolysis temperature, but was lower than raw pellets. This might be attributed to the presence of pores in biocarbons, which might have reduced the mechanical properties of the materials. The pyrolysis temperature increase could have decreased the average pore diameters of the biocarbons [75,76,77]. Similar results were obtained by Xing et al. (2018), who recorded a decrease in durability of corn (maize) straw pellets after pyrolysis, at an even lower value of 31.82% [47].

The content of heavy metals in solid biofuels is important in terms of environmental protection, durability of boilers and the possibility of ash disposal. The chemical properties of biomass have a major influence on the composition of pollutant emissions to the atmosphere. New fuels made from a variety of substrates are appearing on the solid biofuels market. This carries the risk of using fuels that will not meet the required standards in terms of heavy metals content. The tested wood pellets did not contain arsenic and cadmium in their composition. Conversely, lead content was found at the average level of 0.18 mg kg^−1^. The use of the pyrolysis process increased the tested parameter to the maximum level of 0.48 mg kg^−1^. The Pb content in raw pellets and obtained biocarbons did not exceed the permissible values specified in the standard [78,79]. According to Zhang et al. (2014), the level of heavy metals emission during the burning of biomass pellets and uncompressed biomass fuels in households was, for gaseous Pb, Cu and Ni, at the levels of 0.03–0.77, 0.47–5.25, and 0.09–0.75 mg kg, respectively. Cd and As were not detected in the gaseous phase [80]. Ilari et al. (2021), in a study on pellet production from residual biomass of greenery, determined the content of arsenic, cadmium and lead at the levels of <1, <0.005, and <1 mg kg^−1^, respectively [81].

Conditions of biomass pyrolysis process, i.e., time and temperature, affect the range of properties of the products used, e.g., the maximum dust explosion pressure. Biocarbon materials obtained from wood pellets had a higher value of this parameter by up to 47% compared with the control samples. This change was related to the increased fragility of biocarbons, higher carbon content and volatile substances. A similar trend was noted in the case of the maximum pressure rise rate, where the highest standard, an average of 19%, was recorded for biocarbons obtained at 500 °C and 15 min. However, it should be noted that the increases recorded from an average level of 288.1 bar s^−1^ were not significant and did not affect the change in dust classification. The pyrolysis process of wood pellets also affected the decrease in the value of the level of the lower explosion limit. A correlation of this parameter with a change in the thermal temperature of wood pellet treatment was recorded. According to the literature, explosion pressure depends mainly on the content of cellulose and lignin, and the rate of pressure increase is influenced by the content of hemicellulose. It has also been reported that the volatile dust content, size and shape of biomass particles directly affect the threat of ignition and dust explosion [65,82,83,84]. Studies conducted by Shelf (2020) indicated that the maximum explosion pressure of coconut dust did not exceed 7 bar [85]. In turn, Zhao (2019) stated that this parameter for coal dust was a maximum of 7.7 bar, and 7.3 bar for wood dust [66].

The use of the pyrolysis process influenced an increase in the value of the explosivity index, Kst max, relative to the control samples. These changes did not affect the classification of the obtained biocarbons in the context of explosiveness susceptibility, and all analyzed materials were included in class St1—a material not susceptible to explosiveness. A maximum value of this parameter at 94.75 bar s^−1^ was recorded for biocarbons obtained from coniferous pellets (pyrolysis 500 °C and 15 min). The maximum explosion pressure for all analyzed materials was recorded within 200 ms of the time of the initiation. As reported by Saeed et al. (2016), crop residues, i.e., bagasse and wheat straw with a high ash content, were characterized by an index of Kst max at 103 and 82 bar s^-1^, respectively [86]. The value of this parameter for coal and wood dust was, in turn, at the level of 85 and 104 bar s^−1^, respectively [66]. Studies carried out on dust from waste biomass of maize cobs and peanut shells determined this parameter was in the range of 25–60 bar s^−1^ and were not less than for coal, which reflected its lower calorific value [87]. Sensitivity and severity of dust explosions increased with the increase in the concentration of dust, oxygen, and combustible agents, and with the decrease in dust particle size, moisture, and inertants [88].

## 5. Conclusions

Using the pyrolysis process to produce high-energy biocarbons from wood pellets can provide a beneficial and attractive solution in terms of energy utility, and, to some extent, energy security, as a low-carbon energy source.

The results of the analyses of the impact of the pyrolysis process of wood pellets on the basic energy properties of the obtained biochars indicate that this method can significantly increase the calorific value of fuel without reducing the safety of its processing. For the three types of wood pellets tested, i.e., oak, coniferous and mixed pellets (calorific value below 20 MJ kg^−1^), the maximum increase in calorific value after the pyrolysis process was observed to be 30.45, 31.49, and 30.73 MJ kg^−1^, respectively. There was also a positive correlation between total carbon content, ash and temperature, and the duration of the pyrolysis process. Thermal valorization of wood pellets also produced a statistically significant decrease in volatile substance content several times. There was a decrease in the durability of pellets after thermal treatment, which did not significantly reduce the quality of the obtained biofuels. The results did not find an elevated content of heavy metals in the tested materials. However, there were no changes in the classification of explosiveness of biocarbon dust relative to the biomass of non-thermally processed pellets. The mean explosivity index, Kst max value, for all wood pellets tested was 78.07 bar s^−1^, while for biocarbons it was 87.04 bar s^−1^.

The analyses show that the pyrolysis process can be used to valorize wood pellets to produce high quality biocarbon materials. The studies also provide information regarding the effects of pyrolysis carried out after previous biomass pelletization. Biocarbon materials obtained in this way are characterized by a high calorific value with simultaneous safety of production and use. The application of high quality biocarbon materials in the energy industry could become typical particularly for small installations, often home solutions, which do not have an extensive technological line. The results necessitate further research in this direction, to gain more extensive knowledge regarding the safety principles of production, storage, transport and use of these new fuels.

## Figures and Tables

**Table 1:** Dust explosion classes. Copyright European Standards, 2011.

Explosion Class	K st Max Value [Bar s^−1^]
St1	≤200
St2	200–300
St3	>300

**Table 2:** Parameters analyzed with research methods.

Parameter	Research Method
Content of carbon, nitrogen and hydrogen	PN-EN 15104:2011 [48]
Ash content	PN-EN 13775:2010 [49]
Content of volatile substances	PN-EN 15138:2011 [50]
Calorific value	PN-EN 13918:2010 [51]
Mechanical durability	PN-EN 17831-1:20169-02 [52]
Maximum explosion pressure	PN-EN 14034-1 [53]
Maximum rate of pressure rise	PN-EN 14034-2 [54]
Explosion index Kst max	PN-EN 14034-2 [54]
Lower explosion limits	PN-EN 14034-3 [55]

**Table 3:** The content of general nitrogen, total carbon, hydrogen, ash, volatile substances, heavy metals and durability, and calorific value, of oak sawdust pellets and biocarbons.

	Nitrogen	Carbon	Hydrogen	Ash	Volatile Substances	Durability	Calorific Value	Heavy Metals		
								As	Cd	Pb
	%						MJ kg^−1^	mg kg^−^^1^		
OP0	<0.04	51.46 ^a^ ± 0.18	5.96 ^d^ ± 0.03	0.53 ^a^ ± 0.04	81.73 ^c^ ± 0.05	99.12 ^c^ ± 0.22	18.27 ^a^ ± 0.09	<0.01	<0.01	0.11 ^a^ ± 0.00
OP1		75.25 ^b^ ± 0.37	4.33 ^c^ ± 0.03	3.75 ^b^ ± 0.02	37.05 ^b^ ± 0.04	44.92 ^a^ ± 0.11	27.22 ^b^ ± 0.09			0.16 ^b^ ± 0.01
OP2		77.72 ^bc^ ± 0.43	4.16 ^c^ ± 0.02	3.81 ^b^ ± 0.03	36.12 ^b^ ± 0.12	45.16 ^a^ ± 0.28	28.20 ^b^ ± 0.11			0.15 ^b^ ± 0.01
OP3		77.97 ^bc^ ± 0.23	4.27 ^c^ ± 0.02	4.48 ^c^ ± 0.07	35.35 ^b^ ± 0.15	45.34 ^a^ ± 0.16	28.35 ^b^ ± 0.06			0.15 ^b^ ± 0.02
OP4		75.63 ^bc^ ± 0.08	3.83 ^b^ ± 0.02	5.55 ^d^ ± 0.07	34.76 ^ab^ ± 0.08	51.91 ^b^ ± 0.19	27.62 ^b^ ± 0.11			0.23 ^c^ ± 0.01
OP5		78.59 ^bc^ ± 0.12	3.82 ^b^ ± 0.01	6.31 ^e^ ± 0.06	32.63 ^ab^ ± 0.13	52.11 ^b^ ± 0.23	29.25 ^b^ ± 0.15			0.21 ^c^ ± 0.01
OP6		79.21 ^bc^ ± 0.12	3.77 ^b^ ± 0.01	6.59 ^e^ ± 0.06	31.07 ^ab^ ± 0.13	52.28 ^b^ ± 0.44	29.64 ^b^ ± 0.12			0.24 ^c^ ± 0.02
OP7		75.59 ^bc^ ± 0.09	3.50 ^a^ ± 0.01	5.75 ^d^ ± 0.05	30.89 ^a^ ± 0.10	55.67 ^b^ ± 0.31	29.29 ^b^ ± 0.16			0.32 ^d^ ± 0.02
OP8		81.27 ^c^ ± 0.06	3.46 ^a^ ± 0.02	5.82 ^d^ ± 0.02	29.39 ^a^ ± 0.09	55.88 ^b^ ± 0.18	29.81 ^b^ ± 0.09			0.35 ^d^ ± 0.01
OP9		81.41 ^c^ ± 0.20	3.19 ^a^ ± 0.01	6.63 ^e^ ± 0.06	28.34 ^a^ ± 0.10	56.14 ^b^ ± 0.42	30.45 ^b^ ± 0.16			0.34 ^d^ ± 0.02

Differences between average values marked with the same Arabic letters (a–e) are not statistically significant at the level of *p* ≤ 0.05, according to the Duncan test.

**Table 4:** The content of general nitrogen, total carbon, hydrogen, ash, volatile substances, heavy metals and durability, calorific value of coniferous sawdust pellets and biocarbons.

	Nitrogen	Carbon	Hydrogen	Ash	Volatile Substances	Durability	Calorific Value	Heavy Metals		
								As	Cd	Pb
	%						MJ kg^−1^	mg kg^−^^1^		
OP0	<0.04	53.25 ^a^ ± 0.18	6.21 ^d^ ± 0.02	0.37 ^a^ ± 0.07	82.81 ^c^ ± 0.15	98.54 ^c^ ± 0.21	19.31 ^a^ ± 0.08	<0.01	<0.01	0.23 ^a^ ± 0.01
OP1		75.98 ^b^ ± 0.05	4.49 ^c^ ± 0.02	3.16 ^b^ ± 0.07	39.62 ^b^ ± 0.15	44.34 ^a^ ± 0.19	28.88 ^b^ ± 0.08			0.32 ^b^ ± 0.01
OP2		78.86 ^bc^ ± 0.13	4.32 ^c^ ± 0.04	3.54 ^bc^ ± 0.08	39.61 ^b^ ± 0.11	44.62 ^a^ ± 0.14	29.36 ^b^ ± 0.07			0.31 ^b^ ± 0.02
OP3		80.21 ^bc^ ± 0.21	4.17 ^c^ ± 0.03	3.89 ^c^ ± 0.04	38.01 ^ab^ ± 0.16	45.11 ^a^ ± 0.32	29.41 ^b^ ± 0.07			0.34 ^b^ ± 0.01
OP4		80.48 ^bc^ ± 0.13	3.61 ^b^ ± 0.02	3.70 ^c^ ± 0.06	36.77 ^ab^ ± 0.11	51.44 ^b^ ± 0.27	29.66 ^b^ ± 0.08			0.39 ^c^ ± 0.01
OP5		80.96 ^bc^ ± 0.15	3.60 ^b^ ± 0.03	3.75 ^c^ ± 0.03	35.51 ^ab^ ± 0.13	51.59 ^b^ ± 0.41	29.85 ^b^ ± 0.06			0.38 ^c^ ± 0.01
OP6		83.00 ^bc^ ± 0.13	3.40 ^ab^ ± 0.01	4.28 ^d^ ± 0.04	33.14 ^ab^ ± 0.07	51.96 ^b^ ± 0.28	30.07 ^b^ ± 0.05			0.40 ^c^ ± 0.01
OP7		81.99 ^bc^ ± 0.18	3.25 ^a^ ± 0.02	4.22 ^d^ ± 0.05	31.28 ^ab^ ± 0.08	55.43 ^b^ ± 0.23	30.85 ^b^ ± 0.09			0.47 ^d^ ± 0.01
OP8		84.32 ^c^ ± 0.02	3.21 ^a^ ± 0.02	4.31 ^d^ ± 0.04	30.47 ^a^ ± 0.07	55.75 ^b^ ± 0.33	31.26 ^b^ ± 0.08			0.48 ^d^ ± 0.02
OP9		85.21 ^c^ ± 0.11	3.02 ^a^ ± 0.01	4.73 ^d^ ± 0.05	29.24 ^a^ ± 0.09	55.98 ^b^ ± 0.22	31.49 ^b^ ± 0.03			0.48 ^d^ ± 0.02

Differences between average values marked with the same Arabic letters (a–d) are not statistically significant at the level of *p* ≤ 0.05 according to the Duncan test.

**Table 5:** The content of general nitrogen, total carbon, hydrogen, ash, volatile substances, heavy metals and durability, calorific value of coniferous and deciduous pellets and biocarbons.

	Nitrogen	Carbon	Hydrogen	Ash	Volatile Substances	Durability	Calorific Value	Heavy Metals		
								As	Cd	Pb
	%						MJ kg^−1^	mg kg^−^^1^		
OP0	<0.04	52.52 ^a^ ± 0.12	6.16 ^d^ ± 0.03	0.31 ^a^ ± 0.05	82.66 ^c^ ± 0.13	98.87 ^c^ ± 0.21	19.13 ^a^ ± 0.04	<0.01	<0.01	0.21 ^a^ ± 0.01
OP1		75.67 ^b^ ± 0.20	4.49 ^c^ ± 0.04	2.71 ^b^ ± 0.06	38.51 ^b^ ± 0.11	44.51 ^a^ ± 0.13	27.86 ^b^ ± 0.09			0.29 ^b^ ± 0.00
OP2		78.47 ^b^ ± 0.11	4.37 ^c^ ± 0.03	2.95 ^b^ ± 0.05	38.04 ^b^ ± 0.06	44.94 ^a^ ± 0.24	28.55 ^b^ ± 0.08			0.30 ^b^ ± 0.02
OP3		79.16 ^b^ ± 0.14	4.30 ^c^ ± 0.03	3.46 ^bc^ ± 0.05	36.83 ^ab^ ± 0.08	45.28 ^a^ ± 0.16	28.71 ^b^ ± 0.11			0.29 ^b^ ± 0.02
OP4		78.11 ^b^ ± 0.10	3.82 ^b^ ± 0.02	3.89 ^bc^ ± 0.07	35.94 ^ab^ ± 0.12	51.63 ^b^ ± 0.42	28.42 ^b^ ± 0.11			0.36 ^c^ ± 0.01
OP5		79.84 ^bc^ ± 0.07	3.79 ^b^ ± 0.02	4.30 ^c^ ± 0.06	34.24 ^ab^ ± 0.04	51.86 ^b^ ± 0.26	29.37 ^b^ ± 0.07			0.36 ^c^ ± 0.02
OP6		81.17 ^bc^ ± 0.08	3.69 ^ab^ ± 0.02	4.70 ^c^ ± 0.06	32.29 ^ab^ ± 0.09	52.06 ^b^ ± 0.21	29.71 ^b^ ± 0.03			0.38 ^c^ ± 0.01
OP7		78.84 ^b^ ± 0.07	3.48 ^a^ ± 0.04	4.25 ^c^ ± 0.05	31.22 ^ab^ ± 0.09	55.6 ^b^ ± 0.33	29.93 ^b^ ± 0.15			0.43 ^d^ ± 0.02
OP8		82.87 ^c^ ± 0.03	3.43 ^a^ ± 0.05	4.35 ^c^ ± 0.01	30.08 ^a^ ± 0.05	55.68 ^b^ ± 0.39	30.34 ^b^ ± 0.07			0.44 ^d^ ± 0.02
OP9		83.32 ^c^ ± 0.09	3.24 ^a^ ± 0.02	4.95 ^c^ ± 0.07	28.97 ^a^ ± 0.11	56.07 ^b^ ± 0.28	30.73 ^b^ ± 0.11			0.44 ^d^ ± 0.01

Differences between average values marked with the same Arabic letters (a–d) are not statistically significant at the level of *p* ≤ 0.05 according to the Duncan test.

**Table 6:** Level of lower explosive limit, maximum pressure rise rate, maximum explosion pressure of dust from oak, coniferous, mixed pellets and their pyrolysates.

Material	Pmax	(dp/dt)max	LEL—Lower Explosion Limit
	Bar	bar s^−1^	g m^3^
OP0	7.78	282.39	750
OP1	8.21	289.74	
OP2	8.54	294.38	500
OP3	8.86	302.16	
OP4	9.29	307.24	
OP5	9.94	320.54	
OP6	10.06	323.26	
OP7	10.35	327.77	
OP8	10.96	332.40	
OP9	11.15	335.97	
CP0	8.10	293.88	750
CP1	8.55	301.53	
CP2	8.88	306.36	
CP3	9.22	314.46	500
CP4	9.67	319.75	
CP5	10.35	333.58	
CP6	10.47	336.41	
CP7	10.77	341.10	
CP8	11.40	345.93	
CP9	11.60	349.64	
MP0	7.94	288.01	750
MP1	8.38	295.50	
MP2	8.71	300.24	
MP3	9.04	308.18	
MP4	9.48	313.36	500
MP5	10.14	326.92	
MP6	10.26	329.69	
MP7	10.56	334.29	
MP8	11.18	339.02	
MP9	11.37	342.66	

## Data Availability

Not applicable.
